# Maternal characteristics and birth outcomes resulting from births before arrival at health facilities in Nkangala District, South Africa: a case control study

**DOI:** 10.1186/s12884-017-1580-5

**Published:** 2017-12-02

**Authors:** Sikhulile Khupakonke, Andy Beke, Donald H. A. Amoko

**Affiliations:** School of Health Systems and Public Health, University of Pretoria, Pretoria, South Africa

**Keywords:** Birth before arrival, Out-of-hospital births, Maternal and neonatal outcomes, Hospital delivery, Nkangala, South Africa

## Abstract

**Background:**

Risks of severe, avoidable maternal and neonatal complications at birth are increased if the birth occurs before arrival at the health facility and in the absence of skilled birth attendants. Birth Before Arrival (BBA) is a preventable phenomenon still common in modern-day practice despite extensive improvements made in obstetric care and in accessibility to healthcare in South Africa. This study aimed to determine the risk factors and outcomes in mothers and babies associated with being born before arrival at hospitals.

**Methods:**

A prospective case control study design was conducted. All BBAs presenting to the hospitals in Nkangala District between November 2015 and February 2016 were included and compared to a consecutive hospital delivery occurring immediately after the arrival of each BBA. T-tests and chi square tests were used to analyse the differences between the groups and a binary logistic regression analysis used to determine predictors of BBAs. All statistical analysis were done using STATA version 14 using a 5% decision level and a 95% confidence interval.

**Results:**

During the study period, 4397 in-facility births and 201 BBAs were recorded, 78 BBAs and 75 controls were investigated in this study. The district BBA prevalence was 4.6%. Risk factors identified in mothers of BBAs were: single mothers (83.3% vs 69.3%; *p* = 0.04); residing in an informal settlement (23.1% vs 5.3%; *p* = 0.002); and higher gravidity with plurigravida significantly more (60.3% vs 32.5%; *p* < 0.0001). A prevalent maternal complication in cases was haemorrhage due to retained placenta. Most neonates were born alive with a higher proportion of cases experiencing perinatal complications such as respiratory distress, hypothermia and asphyxia. No significant differences in maternal age, employment status and immediate birth outcomes were found. Residing in informal settlements, higher gravidity, unplanned pregnancy, low birth weight and unbooked were found to predict the occurrence of BBAs.

**Conclusion:**

Although no significant numbers of mortalities were recorded in this study, service delivery interventions targeting the reduction of BBAs are needed so as to minimise the morbidity experienced by the group.

## Background

Births before arrivals (BBAs) still occur at alarming rates especially in low-middle income countries like South Africa; even with extensive improvements made in general access to healthcare post the apartheid era [1, 2]. “BBA” is a common term referring to births that occur either at home or in transit before the mother reaches a healthcare facility. Most of these births occur without the supervision of skilled health personnel, with such circumstances exposing both the mother and baby to various complications that could have been avoided if the birth had occurred in a healthcare facility [3]. BBAs constitute a special group of people at risk of significant adverse maternal and neonatal outcomes contributing to morbidity and mortality experienced in obstetric care [3]. UNICEF estimates that globally every year, 4 million babies die in the first 4 weeks of life (the neonatal period) which is closely linked to half a million maternal deaths and about 4 million stillbirths [4, 11]. It is also alarming that three-quarters of neonatal deaths happen in the first week and the highest risk of death being on the first day of life [4].

South Africa reports high overall levels of utilisation of maternal health services, with 92% of women reporting one or more ANC visits and 91% having skilled birth attendants at delivery [5]. An analysis of rising global child deaths (Child Mortality Rates) noted an increase in neonatal deaths exacerbated by the high number of births occurring outside the health facilities, which are more than half in some countries, despite an increase in the number of institutional deliveries worldwide [6]. The documentation of BBAs is still lacking; developed countries record an incidence between 0.1% - 0.3%, which rises exponentially in low income countries to greater that 50% in countries such as India and Ethiopia [2]. The rate of BBAs serve as an index of accessibility to perinatal care [7]; a rate greater than 1.5% signals challenges in health care provision. If such a rate exists, further investigations and appropriate interventions are merited [7]. According to the 2015 Demographic Health Information System (DHIS) statistics, the BBA prevalence of South Africa is currently at 6.33% and thus requires an array of investigations that will enable understanding of the contributing factors [8].

The literature acknowledges significant mortality and morbidity in both mothers and babies associated with babies BBA. Risk factors associated with BBAs include pregnant women with high parity, low education, un-booked at the facility and with no ANC visits [2, 3, 7, 9]. Most recorded maternal morbidity associated with BBAs were postpartum haemorrhage due to uterine atony, retained placenta and products of conception (POC), obstetrical lacerations and tears, and puerperal sepsis [2, 4, 9–11]. The 3 delay model and the Perinatal Problem Identification Program (PPIP) are quality-improvement tools used to identify and understand gaps in access to adequate management of obstetric emergencies [12]. These quality-improvement tools found that patient-related factors contributed 45.9% to the number of maternal deaths. The delay in seeking medical attention during labour accounted for 4.9% of all neonatal deaths in the country, as shown by the 2010 and 2011 Saving Babies report [12]. The commonly documented mortality and morbidity associated with BBAs in neonates result from hypothermia, low birth weight (LBW), hypoglycaemia, pre-maturity, respiratory distress, neonatal sepsis and asphyxia [2, 3, 9, 10, 12, 13]. During birth, it is estimated that about 5% - 10% of new-borns need some assistance with breathing, with 1% requiring extensive resuscitation [4, 12].

Studies worldwide have demonstrated that obstetric complications during birth occur, even in patients presenting with no known risks, making the presence of adequately skilled personnel at birth very important in offering the required vital and immediate care to the mother and baby. In this way, the skilled personnel alleviates direct causes of maternal and neonatal mortality [4]. This study aimed to establish: i) the characteristics of mothers giving birth before arrival at health facilities; ii) BBA mothers’ reasons for the delay in seeking care; and iii) the effect of BBAs on maternal and neonatal outcomes.

## Methods

An unmatched prospective case control study was conducted in 5 sub-district hospitals from Nkangala District between 1 November 2015 and 29 February 2016. Nkangala district, situated in Mpumalanga Province, has a hospital in each of its 6 sub-districts. This study was conducted in 5 sub-districts, which were Emalahleni, Steve Tswete, Thembisile Hani, Dr. J.S. Moroka and Victor Khanye. A case (BBA) was defined as any baby born either at home or en-route to hospital before the mother arrived at the maternity ward within 24 h of delivery and whose delivery was not assisted by trained health professionals such as a medical practitioner, registered midwife, registered nurse or paramedics. The controls were babies born by normal vaginal delivery in the hospital, immediately after the arrival of each BBA. Exclusions included births by caesarean section, multiple deliveries and professionally assisted deliveries occurring at home, in an ambulance or at casualty. Identified cases and controls were approached for inclusion in the study at the post-natal ward. Informed consent was obtained, after which a structured questionnaire was administered. Clinical records were also reviewed to collect and verify medical information.

A researcher-designed questionnaire was used to capture information on each participants’ socio-economic status, previous pregnancies, current pregnancy and the neonate. All variables measured were informed by an extensive literature search conducted on risk factors influencing the occurrence of BBAs. All data was captured on EpiInfo 7 and analysed using STATA 14. Data cleaning and consistency checks were performed before analysis. Exploratory data analysis, which consisted of basic univariable and bi-variable analysis, was conducted on key variables including demographic data. Bi-variable comparisons were performed using t-tests for continuous variables and chi-squared tests for categorical variables. Descriptive statistics in the form of frequency distribution tables were used to evaluate the reasons for delay given by BBA mothers. Binary logistic regression was used to identify maternal and neonatal variables independently associated with being a BBA. Those variables which were significant at the univariable analysis were entered into a multiple logistic regression using a forward and backward selection procedure. Model of best fit was determined by the log-likelihood estimate. For all statistical analyses, a 5% level of significance and a 95% confidence interval were used.

## Results

**Fig. 1:**
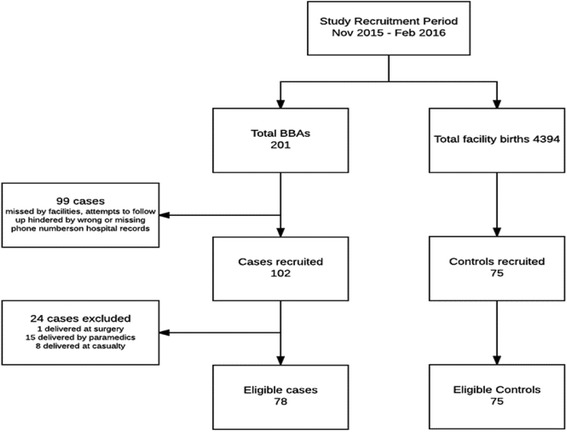
Study recruitment flow diagram

During the study period, 201/4394 (4.6%) of babies were born before arrival to the hospital (BBA). The BBA prevalence stated above was obtained by dividing the total number of BBAs by the total number of in-facility births i.e. livebirths, stillbirths and BBAs. Although there were 201 BBAs, as illustrated in Fig. 1 above, the study only captured 102 BBAs and 75 controls. The discrepancy resulted from a lack of opportunities to interview cases before they were discharged from the hospital. Maternity personnel, used as data collectors in this study, are understaffed and often overwhelmed by the high number of deliveries on a daily basis. Twenty four (24) BBAs had assisted delivery by skilled personnel and hence were not eligible for inclusion. This study therefore presents results for 78 cases and 75 controls.

Table 1 above portrays the overall characteristics of the two groups. As regard the mother’s social status, the mean maternal age was similar in both groups (27 years BBA group v 26 years control group). Although slightly higher for BBA mothers, the difference was not statistically significant (*p* = 0.25). The majority of the women in the study (82%), were aged between 18 and 35 years of age while 15 women (19.7%) were below the age of 18 years (6 BBA group Vs 9 control group) and 17% were above 35 years of age (7.7% BBA group Vs 9.9% control group). Significantly, BBA mothers were most likely to be single (83.3% vs 69.3%: OR = 2.2) and reside in an informal settlement (23.1% vs 5.3%: OR = 5.3). There were no significant differences between groups in terms of employment status, level of education and access to a means of communication (cell phone). In assessing the maternal history (previous pregnancies) of study participants, BBA mothers were found to be of significantly higher gravidity, with (60.3% vs 32.5%) pregnant for more than 3 times (plurigravida). BBA mothers had a history of BBAs in previous pregnancies (20.5% vs 4%: OR = 6.2, *p* = 0.003) and had experienced statistically similar deaths in previous pregnancies or babies (*p* = 0.22).

**Table 1:** Overall characteristics of cases and controls

	BBAs	Controls	OR	*p-value*
	*N* = 78	*N* = 75	CI = 95%	
Mother’s social status				
Age (years)				
Mean	27	26	–	0.25
Groups’ *n* (%)			–	0.60
< 18	6 (7.7)	9 (12.0)		
18–35	66 (84.6)	59 (78.7)		
> 35	6 (7.7)	7(9.3)		
Marital status *n* (%)			2.2	0.0413
Married	13 (16.7)	23 (30.7)		
Single	65 (83.3)	52 (69.3)		
Highest Education *n* (%)			–	0.18
Below tertiary	71 (91.0)	62 (83.0)		
Tertiary	7 (8.9)	12 (16.0)		
Employment status *n* (%)	*N* = 78	*N* = 74	–	0.35
Unemployed	61 (78.2)	53 (71.6)		
Employed	17 (21.8)	21 (28.4)		
^a^Access to cell phone *n* (%)			–	0.95
Yes	74 (94.9)	71 (94.7)		
No	4 (5.1)	4 (5.3)		
^a^Type of housing *n* (%)			5.3	0.002
Formal settlement	60 (76.9)	71 (90.7)		
Informal settlement	18 (23.1)	4 (5.3)		
Previous Pregnancies				
Gravidity *n* (%)	*N* = 78	*N* = 74	–	< 0.0001
Primigravida	8 (10.2)	28 (37.8)		
Multigravida	23 (29.5)	22 (29.7)		
Grand multigravida	47 (60.3)	24 (32.5)		
History of BBAs *n* (%)			6.2	0.003
Yes	16 (20.5)	3 (4.0)		
No	62 (79.5)	72 (96.0)		
Mortality in previous births *n* (%)			–	0.22
None	65 (83.3)	65 (86.7)		
Yes	13 (16.7)	10 (13.3)		
Miscarriage	5 (6.4)	2 (2.6)		
Still births	3 (3.9)	6 (8.1)		
Child deaths	5 (6.4)	2 (2.6)		
Present Pregnancy				
Reaction to index pregnancy *n* (%)	*N* = 77	*N* = 75	3.1	0.002
Planned	17 (22.1)	34 (46.6)		
Unplanned	60 (77.9)	39 (53.4)		
Antenatal Care (ANC)				
^a^Booking status n (%)			7.3	0.005
Booked)		65 (83.3)	73 (97.3)	
Unbooked	13 (16.7)	2 (2.7)		
Number of visits				
Mean (SD)	3 (2.3)	4 (2.2)	–	0.002
Groups’ *n* (%)	*N* = 65	*N* = 73	3.1	0.006
< 5 times	60 (92.3)	58 (79.5)		
> 5 times	5 (7.7)	15 (20.5)		
Gestation at booking (weeks)	*N* = 65	*N* = 73	–	0.041
Mean (SD)	23 (8.4)	20 (6.4)		
Comorbidities *n* (%)	*N* = 77	*N* = 75		
HIV positive	23 (29.9)	20 (26.7)	–	0.66
^a^Hypertensive	5 (6.5)	2 (2.7)	–	0.33
Duration of labour (minutes)	*N* = 76	*N* = 75	–	<0.0001
Mean (SD)	241.4 (359)	556.2 (483)		
Mode of transport used *n* (%)	*N* = 77	*N* = 75	–	–
Walked	0 (0)	1 (1.3)		
Public transport	3 (3.9)	5 (6.7)		
Private vehicle	19 (24.7)	33 (44.0)		
Ambulance	55 (71.4)	36 (48.0)		
Ambulance reaction time (minutes)	*N* = 36	*N* = 6	–	–
Median [Range]	111 [15; 215]	106 [30; 195]		
Place of delivery *n* (%)				
Home	73 (93.6)	–		
In transit	5 (6.4)	–		
Maternity ward	–	75 (100)		
Adverse maternal outcomes *n* (%)			–	0.43
None	44 (56.4)	47 (62.7)		
Yes	34 (43.6)	28 (37.3)		
Haemorrhage due to-:				
Uterine atony	0 (0)	1 (1.3)		
Retained placenta	16 (20.5)	3 (4.0)		
Obstetric tears	18 (23.1)	24 (32.0)		
Neonate				
^a^Birth outcomes *n* (%)	*N* = 77	*N* = 75	–	0.98
Alive	74 (96.1)	73 (97.3)		
Still born	2 (2.6)	2 (2.7)		
Died after delivery	1 (1.3)	0 (0)		
Birth weight (grams)	*N* = 76	*N* = 74	–	0.011
Mean	2689	2995		
(IQR)	(2300, 3200)	(2795, 3392)		
Groups’ *n* (%)			2.7	0.011
LBW <2500	26 (34.2)	12 (16.2)		
Normal >2500	50 (65.8)	62 (83.8)		
Gender *n* (%)			–	0.52
Male	40 (51.9)	35 (46.7)		
Females	37 (48.1)	40 (53.3)		
Gestation at delivery (weeks)	*N* = 71	*N* = 72	3.4	0.003
Preterm <36	25 (35.2)	10 (13.9)		
Term 36–41	46 (64.8)	62 (86.1)		
^a^Perinatal complications *n* (%)	*N* = 69	*N* = 73	7.7	<0.0001
Yes	19 (27.5)	3 (4.1)		
No	54 (72.5)	66 (95.9)		
^a^Intervention n (%)			–	0.34
ICU admission	5 (6.4)	1 (1.3)		
Nursery admission	70 (89.7)	72 (96.0)		
Duration of admission (days)				
Mean (SD)	2.61 (3.31)	1.44 (1.56)	–	0.0061

T-test and Chi-squared tests were used where appropriate, ^a^Fisher’s exact test was used, *SD* Standard Deviation, *CI* Confidence Interval, *OR* Odds Ratio

Regarding the index pregnancy, most women (65.1%) referred to the current pregnancy as unplanned, with BBA mothers significantly more (77.9% *p* = 0.002). A higher proportion of BBA mothers were unbooked for antenatal care (16.7% vs 2.7%: OR = 7.3, *p* = 0.005). The mean gestational age at booking was significantly higher in BBA mothers (23 weeks Vs 20 weeks, *p* = 0.04). Generally poor antenatal care attendance applied to most women (85.6%), who attended ANC classes less than 5 times during the pregnancy, although BBA mothers were significantly more (51%Vs 49%: OR = 3.1, *p* = 0.006). BBA mothers also experienced a shorter duration of labour compared to their counterparts with a mean of 241 (SD = 359) minutes vs 556 (SD = 483) minutes. The most common mode of transport used was the ambulance (71.4% cases Vs 48% controls) and the reaction time slightly more in cases than controls (111 min vs 106 min). No significant differences in the morbidity of mothers in either group were found; comorbid factors that were similar for BBA mothers and controls were HIV positivity (29.9% Vs 26.7%, *p* = 0.66) and hypertension (6.5% Vs 2.7%, *p* = 0.33). Although significantly few mothers (40.5%) experienced adverse maternal outcomes, a higher proportion of BBA mothers encountered obstetric haemorrhages due to retained placenta (20.5%).

The immediate birth outcomes in neonates were comparable; most babies were alive at birth (96.7%), 4 babies were still born (2 BBA and 2 control) and 1 BBA died after delivery. The mean birthweight for cases was significantly lower at 2689 g (95% CI 2511 g - 2869 g) than 2996 g (95% CI 2844 g – 3148 g) for controls. BBAs were more likely to be LBW babies, weighing less than 2500 g (34.2% vs 16.2%: OR = 2.7, *p* = 0.011). There were more males (51.9%) in the cases and more females (53.3%) in the controls but the differences were not significant (*p* = 0.52). Most BBAs were born pre-term (35.2% vs13.9%; OR = 3.4 pvalue = 0.003), experienced perinatal complications (27.5% vs 4.1%: OR = 7.7, pvalue =0.034) and had a lengthier mean hospital stay of 3 days (SD = 3.31) v 1 day (SD = 1.56) than control babies. No significant differences in ICU admission rates existed for cases and controls (pvalue = 0.34**).**

### BBA mothers’ reasons for delay in seeking care

**Fig. 2:**
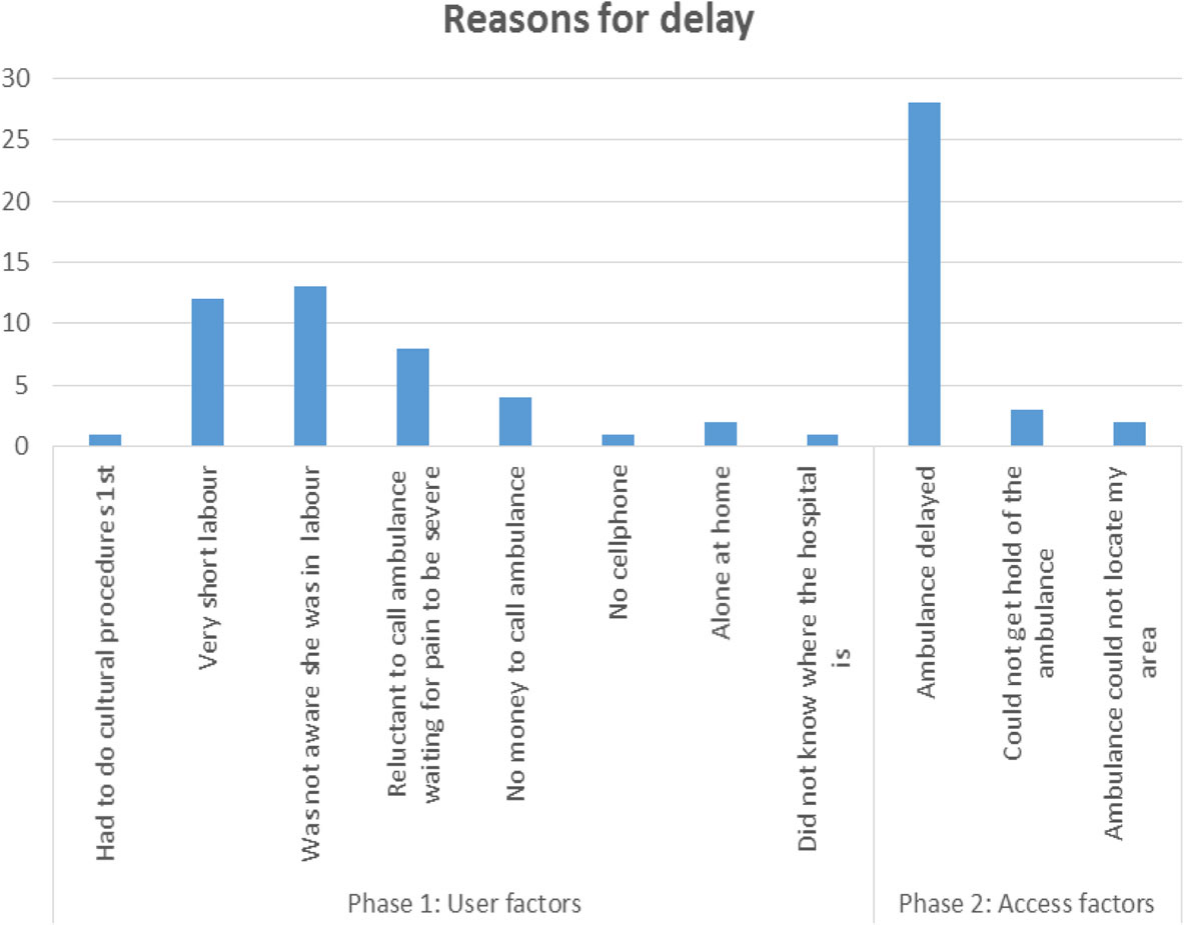
The Three Delay model

Fig. 2 below illustrates the 3 delay model which was used to investigate reasons for the BBA mothers’ delay in seeking care early in labour [12]. Reasons were classified according to 3 phases:

- Phase 1: Delay in decisions to seek care associated with the user
- Phase 2: Delay as a result of issues relating to accessibility of services
- Phase 3: Delay in receiving adequate health care.

The most common reason in Phase 1 of the model included people who were unaware that they were in labour (31%), experienced short labour (29%) and were reluctant to call the ambulance while they waited for either the water to break or pains to be severe (19%) among other reasons. Phase 2 was mostly characterised by the delay of the ambulance when called (84%) or failure to reach the ambulance from a cell phone (9%). Two cases (6%) struggled to find the location of the hospital while in labour. No reasons given were related to Phase 3: service delivery.

### Predictors of having a BBA

A logistic regression analysis to establish predictors of the occurrence of BBAs yielded the following results, set out in Table 2 below. In univariable analysis, maternal characteristics that were found to be associated with the occurrence of a BBA were single (not married), residing in an informal settlement, higher gravidity, having an unplanned pregnancy and previous history of BBAs. Other associated risk factors included being unbooked at ANC and experiencing a short duration of labour, preterm delivery and an LBW baby. In multivariable analysis, residing in an informal settlement (OR = 7.6; 95% CI 1.43–40.33), higher gravidity (OR = 1.41; 95% CI 0.48–4.16), an unplanned pregnancy (OR = 3.06; 95% CI 1.18–7.90), and unbooked (booked: OR = 0.082; 95% CI 0.03–1.23) were risk factors. A short duration of labour (highest duration: OR = 0.08; 95% CI 0.02–0.29) and having an LBW baby (normal weight: OR = 0.27; 95% CI 0.09–0.83) were also associated with the occurrence of a BBA (see Table 2).

**Table 2:** Logistic regression analysis for predictors of BBAs

For the outcomeBBA	Univariable analysis		Multivariable analysis (final model)*n* = 144		
Co-variable	OR	pvalue	OR	pvalue	95% CI
Settlement					
Formal	1.00	–	1.00	–	–
Informal	5.33	0.001	7.60	0.017	1.43–40.33
Gravidity					
Primigravida	0.19	<0.0001	0.10	0.001	0.02–0.41
Multigravida	1.00	–	1.00	–	–
Grand multigravida	3.16	0.001	1.41	0.530	0.48–4.16
Reaction to pregnancy					
Planned	1.00	–	1.00	–	–
Unplanned	3.08	0.001	3.06	0.021	1.18–7.90
Booking status					
Booked	0.14	0.002	0.20	0.082	0.03–1.23
Unbooked	1.00	–	1.00	–	–
Duration of labour					
< 120 mins	1.00	–	1.00	–	–
120–240 min	2.82	0.011	0.75	0.649	0.22–2.59
241–575 min	0.40	0.019	0.26	0.028	0.08–0.87
> 575mins	0.18	0.000	0.08	0.000	0.02–0.29
Birthweight					
LBW <2500 g	1.00	–	1.00	–	–
Normal >2500 g	0.37	0.011	0.27	0.023	0.09–0.83
Gestation at delivery					
Pre-term	1.00	–			–
Term	0.29	0.003			–
Perinatal complications					
Yes	7.74	0.002			–
No	1.00	–			
Maternal complications					
Yes	1.30	0.430			–
No	1.00	–			–
BBA history					
Yes	6.19	0.001			–
No	1.00	–			–
HIV status					
Positive	1.17	0.661			–
Negative	1.00	–			–
Age categories					
< 18 years	0.61	0.369			–
18–35 years	1.00	–			–
> 35 years	0.81	0.716			–
Marital status					
Married	1.00	–			–
Single	2.21	0.040			–
Education level					
Pre-tertiary)	1.00	–			–
Tertiary	0.51	0.176			–

*OR* Odds Ratio, *n* sample size *CI* Confidence Intervals, *HIV* Human Immunodeficiency Virus

Post regression (final model) demonstrated an area under ROC curve of 0.8803 and the Pearson’s goodness of fit test was statistically non-significant (*p* = 0.9269), indicating a satisfactory goodness of fit for the model. Residuals analysis using m-asymptomatic residuals were also satisfactory. No outliers were seen to exert undue influence on model parameters.

## Discussion

The study obtained a BBA prevalence of 4.6% in the district, over the study period. This figure is comparable to 5.1%, the BBA prevalence of Nkangala obtained from the DHIS database for the year 2015 [8]. The DHIS calculation of the BBA prevalence may be conservative as the BBA indicator used includes only live BBAs. However, from the same database, the BBA prevalence for 2015 in South Africa was 6.33% and 5.24% in Mpumalanga province where Nkangala district had the second highest prevalence rate in the province [8]. The district BBA rate is very high compared to rates obtained in similar studies conducted in South African hospitals: KwaZulu Natal (1.8%) and Gauteng (3%), which is alarming [2, 10]. The district is largely peri-urban, with smaller pockets of peri-rural areas and should have a BBA prevalence comparable to studies conducted in peri-urban settings similar to those mentioned above. Furthermore, the district BBA rate is higher than the index BBA prevalence rate of 1.5%, which is stated to be a measure of accessibility of perinatal services; sufficient to prompt further investigations if exceeded [2, 7].

Maternal factors found to pose a risk of having a BBA were being a single mother, having an unplanned pregnancy and staying in an informal settlement. These factors are consistent with those from other studies. Potter et al. [7] and Chiragdin [3] found poverty and place of residence to be major determinants of BBAs as both factors have a bearing on an individual’s ability to timely access appropriate health services [3, 7]. Residing in informal settlements is usually associated with having a disadvantaged social background, as established by Unterscheider [9] in a BBA study. A disadvantaged background suggests lack of money for: transport, airtime to call EMS and babysitting services for other children when one is in labour. Chiragdin in a similar study in Nairobi found that BBA mothers were likely to be married and housewives contrary to study findings which established BBA mothers to be mostly single [3].

Other social factors such as employment status, maternal age, access to cell phone and low education level were not significantly different between the groups. Al-amoudi [14] and Chiragdin [3] however, found low education level to be a predictor of having a BBA in their study settings. Al-amoudi et al.’s study had rural farming areas [14] as its setting, while Chiragdin’s study was conducted in a patriarchal society, not in favour of womens’ education [3]; which accounts for the differences found. Pertaining to the mothers’ health status, the study established an HIV positivity rate in pregnant women of 28.2% similar to that of the district at 29.6% (HIV prevalence among ANC women, 2011) [15]. There was no significant difference between cases and controls in terms of different comorbidities such as HIV status and hypertension. A study in Dublin found a higher HIV incidence in BBA mothers and established an association between poverty and substance misuse and other infections such as Hepatitis B and C. [9] More studies are needed that aim to establish HIV positivity of the BBAs in the post neonatal period.

Most BBA index pregnancies were unplanned compared to those of controls. This might have had an impact on the mothers’ attitude towards and compliance with attending antenatal classes given at clinics. The study found that BBA mothers were more likely to be unbooked. Booking, if it took place, was after 20 weeks gestation. Those who attended antenatal classes did so fewer times than the WHO-recommended minimum of five ANC visits in each pregnancy [16]. Poor or lack of attendance at ANC is a common risk factor for the occurrence of out-of-facility births [2, 3, 10, 17] that is referred to in the literature. Poor or no attendance leads to a lack of opportunities for health education, birth readiness and emergency preparedness, all of which have the purpose of increasing a mothers’ ability to deal with birth emergencies should they occur [2, 3]. Reasons for delay in seeking help given by BBA mothers included unawareness of indications of labour, reluctance to get help waiting for more severe pains, or waiting for the water to break. Some mothers looked for a hospital only when labour had begun. Ambulance delays are implicated as contributing to the occurrence of BBAs in literature [5, 7, 18]; the differences in ambulance reaction times were prone to recall bias and thus not sufficient to conclude as such in this study however. More research, with a standard approach in recording ambulance reaction times for women in labour, is needed in order to investigate the extent of EMS’s contribution to the BBA problem.

Poverty is seen to play a role in the lack of empowerment of woman and leads to their inability to make decisions to seek help early as shown in the reasons given for the delay. These reasons include lack of airtime to call ambulance, no cell phone, no one at home, or husband being away. Similar reasons for the delay in seeking care early in labour were given in a study done in Nairobi and the delay was attributed to the patriarchal society in which women still relied heavily on men for decision making and financial support [3], which might add to our understanding of such behaviours in a South African context. A study conducted by UNICEF on maternal- and new-born health shows how far reaching the effects of poverty are – from low education attainment, lack of employment to poor living conditions. All these factors disable women’s decision making skills and lead to unwanted pregnancies [4]. The present study has established that a mother’s attitude to the pregnancy influences compliance with antenatal care classes and jeopardises birth preparedness and emergency readiness, which potentially lead to the occurrence of BBAs.

BBA mothers had a higher gravidity and shorter duration of labour than controls. They were also more likely to have a previous history of BBAs than controls were. Gravidity and parity are risk factors for the occurrence of BBAs in most studies [2, 9, 17–19]. Multiparous women are known to experience shorter labours than nulliparous women generally [2, 19]. Harry and Chiragdin found that previous history of BBAs was a predictor for the occurrence of BBAs [3, 17]. Cases and controls showed no difference in mortality for previous babies. This finding seems to suggest a reluctance in cases to have hospital deliveries, as the difference in mortality on previous babies is not significant between the groups. As the cases continue to have more home deliveries with no adverse outcomes that can be associated with the birth, they tend to get comfortable with the phenomenon and fail to see the significance and importance of hospital deliveries. A study by Potter et al. saw a reduction in the BBA rate, which was partly attributed to the women’s experience of deaths in previous off-springs, related to giving birth at home. Mortality in home births encouraged most study controls to give birth in a hospital [7]. A study conducted to assess the access and use of maternal services in South Africa also established that mothers of BBAs were likely to have a past history of BBAs because of staff attitudes and lack of quality service provision in maternity wards [5]. In this study, however, reasons for delay reported by BBA mothers were not sufficient to attribute repeated out-of-facility births to attitudes of staff in maternity wards and poor service delivery.

Immediate birth outcomes for the babies were similar, with most babies born alive and a similar number of still births in both cases and controls. Differences become apparent post-delivery as evident by one BBA who was born alive but died after delivery. BBAs tended to have a lower birthweight and were born preterm compared to controls in this study; these findings are similar to those found by Ballot et al. in a study to determine the survival of low birth weight neonates, that there is a higher likelihood of low birth weight babies born before term [19]. The study argued that the LBW in neonates was more a factor of low gestational age at delivery than the fact that the baby was a BBA. The same study established that being a BBA was a significant determinant of survival in babies with very low birth weights [10, 19]. Additionally, prematurity and low birthweight were found to be consistent findings in various BBA studies as they usually occur as a result of the unanticipated onset of labour [2, 3, 14]. BBAs were more likely to suffer from perinatal complications than controls were, with the most prevalent complications recorded as prematurity, respiratory distress, hypothermia and asphyxia [2, 3, 7, 9, 10].

The literature supports study findings that mothers of BBAs experience more complications than in-facility births, as they have no immediate access to help from skilled health personnel, who have additional assistance from equipment designed to alleviate adverse outcomes should they occur during the birth process. A BBA study done in Rotunda Hospital, Dublin, found BBA babies to experience neonatal morbidity three fold than hospital born babies [9]. There were no maternal deaths recorded in the study in either of the groups; most women did not experience any maternal complications though there were significant differences in complications experienced by BBA mothers compared to controls. The most common complication was a haemorrhage due to retained placenta. These maternal outcomes found in this study support findings in the literature that establish the worldwide leading causes of maternal morbidity associated with BBAs to be: postpartum haemorrhage due to uterine atony; retained placenta; and products of conception (POC). Shock, obstetrical lacerations and tears and puerperal sepsis are additional causes [3, 4, 9, 14, 18].

### Limitations

The major limitation of this study was the use of clinic staff as data collectors. Owing to a chronic shortage of nursing staff coupled with a high number of deliveries daily, clinic staff hardly have sufficient time for additional duties such a study. Extensive training was given on the methods of data collection pertaining to the data-collection instruments used in this study. As BBAs occur any time of the day, the entire unit was involved in the data collection so as to avoid the missing of cases. Missing cases, nonetheless, still occurred as a result of heavy workload and extended working hours the unit staff is exposed to.

This study was a census of all BBAs occurring in the selected facilities for the specified duration and was, therefore, time bound and not powered on obtaining an effect on a statistically determined sample size. This time boundness could be a potential limitation if the obtained sample is not sufficient to detect differences between the groups.

Selection bias was another limitation in this study. The recruitment of cases was sequential while that of the controls was dependent on the time of arrival of a case. Controls were consecutive births occurring immediately after the arrival of each case. The random selection of controls, though dependent on the arrival of a case, was random by the nature of the occurrence of births. When more than two controls were available, selection was randomised by balloting. This procedure was done to minimise potential selection bias.

The study was conducted in 5 major hospitals of Nkangala District. It reflects, therefore, on circumstances of BBA in urban and peri-urban settings with smaller pockets of rural communities and thus does not adequately reflect the true situation in different settings and cannot be generalised. The omission of some district hospitals as study sites was purely the result of logistics, time and budgetary constraints. This omission may further affect the generalisability of study findings to the entire district.

## Conclusion

Though the BBA rate established was lower than the national average, it is still alarmingly high and requires further investigations that will lead to the development of appropriate policies. Predictors of BBAs established in this study were type of human settlement, gravidity, duration of labour, reaction to index pregnancy and ANC attendance. The risk factors identified to be associated with BBAs were mostly related to the social status of the mother such as poverty, as is indicated by the high numbers of single unemployed women, residing in informal settlements and with more than three children to care for. Poverty may be seen as a key driver for the risky sexual behaviour that these women engage in, exposing themselves to sexually transmitted infections and resulting in unplanned pregnancies.

Poverty, higher gravidity and previous history of having BBAs may contribute to the reluctance to attend antenatal classes, which are instrumental in establishing birth and emergency readiness in expectant mothers. Though sufficient medical evidence exists that preterm labour is short and difficult to diagnose, responses of the women on reasons for delay in seeking care were more inclined to some reluctance, which could have emanated from multiple-birth experiences of which some were BBA. Mothers of BBAs experience more complications during birth, with no statistical differences in deaths found in this study.

Women thus need to be empowered both financially and educationally so that they are able to make critical decisions independently, which will enable them to act promptly in cases of emergency. It is paramount to increase knowledge of the health benefits of hospital deliveries and to improve the accessibility and the usage of maternal services such as ANC by reducing poverty-related constraints. This intervention will have an impact on the usage of maternal services and lead to the reduction of maternal, neonatal-mortality and morbidity-related BBAs.

While immediate access-related problems like increasing emergency services coverage may be of importance in the short term, every effort must be employed to minimise adverse outcomes of BBAs and their mothers’ in the long term. A more sustainable approach should be a long-term solution where the importance of assisted delivery in a health care facility is internalised as the desire of every one and thus reinforced through the school curricula to change mind sets from a tender age.

## Abbreviations

ANC: Antenatal Care
BBA: Birth Before Arrival
CMR: Child Mortality Rate
DHIS: District Health Information System
HIV: Human Immune Virus
ICU: Intensive Care Unit
LBW: Low Birth Weight < 2.5 kg
MP: Mpumalanga
POC: Products of Conception
PPIP: Perinatal Problem Identification Program
UNICEF: United Nations International Children’s Emergency Fund
UP: University of Pretoria
WHO: World Health Organisation
